# Retraction: Oncolytic virotherapy promotes radiosensitivity in soft tissue sarcoma by suppressing anti-apoptotic MCL1 expression

**DOI:** 10.1371/journal.pone.0343958

**Published:** 2026-03-04

**Authors:** The PLOS One Editors

After this article [1] was published, concerns were raised about the reported mouse tumor sizes. Specifically:

- Figs 6A appears to report tumor sizes of up to 6,000 mm^3^ with a standard deviation of approximately 800 mm^3^ for the Mock group.
- Fig 6B appears to report tumor sizes of up to 4,200 mm^3^ with a standard deviation of approximately 1,500 mm^3^ in the Mock group.
- The “*in vivo* subcutaneous human STS xenograft tumor models” subsection of the Materials and Methods section states that mice were euthanized when the mean tumor volume reached 4,000 mm^3^, which is higher than what is typically considered acceptable for subcutaneous tumor mouse experiments (2,000–2,500 mm^3^). The article does not provide a rationale for the specified endpoint criteria.

In response to editorial follow-up, the corresponding author stated that mice were euthanized if they had tumors exceeding 4,000 mm^3^ and >20% body weight loss, or if two or more mice had tumors exceeding 4,000 mm^3^ with ≤ 20% body weight loss.

The *PLOS One* Editors remain concerned that the endpoint criteria do not align with internationally accepted animal welfare standards for mouse tumor studies. Therefore, the *PLOS One* Editors retract this article [1].

The ethics approval document number was not reported in [1]. The corresponding author stated the approval numbers from the Ethics Review Committee for Animal Experimentation of Okayama University School of Medicine for the study in [1] are OKU-2018319 and OKU-2018789.

TOmori, HT, YY, SO, JH, KS, TomohiroF, AY, TKunisada, SK, TOzaki, and ToshiyoshiF agreed with the retraction. TKomatsubara and YU either did not respond directly or could not be reached.
